# Filarial Coinfection Is Associated With Higher Bacterial Burdens and Altered Plasma Cytokine and Chemokine Responses in Tuberculous Lymphadenitis

**DOI:** 10.3389/fimmu.2020.00706

**Published:** 2020-04-21

**Authors:** Gokul Raj Kathamuthu, Saravanan Munisankar, Vaithilingam V. Banurekha, Dina Nair, Rathinam Sridhar, Subash Babu

**Affiliations:** ^1^National Institutes of Health-NIRT-International Center for Excellence in Research, Chennai, India; ^2^National Institute for Research in Tuberculosis (NIRT), Chennai, India; ^3^Government Stanley Medical Hospital, Chennai, India; ^4^Laboratory of Parasitic Diseases, National Institute of Allergy and Infectious Diseases, National Institutes of Health, Bethesda, MD, United States; ^5^Frederick National Laboratory for Cancer Research Sponsored by the National Cancer Institute, Frederick, MD, United States

**Keywords:** tuberculosis, lymphadenitis, filariasis, cytokines, chemokines

## Abstract

Filarial infections are known to modulate cytokine responses in pulmonary tuberculosis by their propensity to induce Type 2 and regulatory cytokines. However, very little is known about the effect of filarial infections on extra-pulmonary forms of tuberculosis. Thus, we have examined the effect of filarial infections on the plasma levels of various families of (IL-1, IL-12, γC, and regulatory) cytokines and (CC and CXC) chemokines in tuberculous lymphadenitis coinfection. We also measured lymph node culture grades in order to assess the burden of *Mycobacterium tuberculosis* in the two study groups [Fil+ (*n* = 67) and Fil– (*n* = 109)]. Our data reveal that bacterial burden was significantly higher in Fil+ compared to Fil– individuals. Plasma levels of IL-1 family (IL-1α, IL-β, IL-18) cytokines were significantly lower with the exception of IL-33 in Fil+ compared to Fil– individuals. Similarly, plasma levels of IL-12 family cytokines -IL-12 and IL-23 were significantly reduced, while IL-35 was significantly elevated in Fil+ compared to Fil– individuals. Filarial infection was also associated with diminished levels of IL-2, IL-9 and enhanced levels of IL-4, IL-10, and IL-1Ra. Similarly, the Fil+ individuals were linked to elevated levels of different CC (CCL-1, CCL-2, CCL-3, CCL-11) and CXC (CXCL-2, CXCL-8, CXCL-9, CXCL-11) chemokines. Therefore, we conclude that filarial infections exert powerful bystander effects on tuberculous lymphadenitis, effects including modulation of protective cytokines and chemokines with a direct impact on bacterial burdens.

## Introduction

Lymphatic filariasis is a tropical disease afflicting about 70 million people worldwide (1). It is an infection caused by the nematodes, *Wuchereria bancrofti, Brugia malayi*, and *Brugia timori* and is transmitted by mosquitoes (2). Filarial infections occur predominantly in tropical regions of the world where pulmonary and extra-pulmonary tuberculosis (TB) are co-prevalent (3). Tuberculous lymphadenitis (TBL) is the most common manifestation of extra-pulmonary TB and constitutes 35% of all forms of extra-pulmonary TB (4). The incidence of TBL has increased in parallel with the increase of mycobacterial infections worldwide. Cervical lymphadenopathy is the most common manifestation of TBL but other lymph nodes including inguinal, axillary, mesenteric and mediastinal can be involved (5). The pathogenesis of TBL is still poorly understood (5).

Cytokines are crucial players in host resistance to both pulmonary TB and TBL (6, 7). Thus, Type 1, Type 17 and other pro-inflammatory cytokines (including IL-1, IL-12, and γC family of cytokines) are important for host protection, while Type 2 and regulatory cytokines are detrimental for host immunity against active TB (7). A variety of chemokines are known to play an important role in the immune response to active TB (8, 9). Filarial infections are known to modulate cytokine and chemokine responses in both active and latent tuberculosis (TB) (3). However, the effect of filarial infections on the cytokine and chemokine response to TBL is not known.

Therefore, we aimed to study the cytokine and chemokine profile established during filarial infection to assess its impact on TBL disease. To address this, we measured the plasma levels of different families of (IL-1, IL-12, γC, and regulatory) cytokines and (CC and CXC) chemokines in filaria-TBL coinfection. We show that filarial coinfected individuals have significantly increased bacterial burden in the affected lymph nodes when compared to filarial-uninfected individuals. Our results also show significantly altered cytokine and chemokine responses in filaria-TBL coinfection.

## Results

### Study Population Demographics

The demographics of the study population are listed in (Table 1). There were no significant differences in age and gender between Fil+ and Fil– individuals. Neither age nor gender had any significant effect on the cytokine or chemokine profiles. However, Fil+ individuals had moderately higher bacterial burdens as determined by the culture grade in solid media of the affected lymph node (*p* = 0.045). Hence, filarial coinfection is associated with higher bacterial burdens in TBL.

**Table 1:** Demographics of the TBL individuals.

**Study demographics**	**Fil+**	**Fil–**	***P*-value**
Number of subjects recruited (*n*)	67	109	–
Gender (M/F)	55/12	86/23	–
Median age in years (range)	40.2(18-65)	36.8(19-65)	NS
Culture grade Low bacterial burden (1+) Medium bacterial burden (2+) High bacterial burden (3+)	16 30 21	65 31 13	0.045^a^
Filarial circulating antigen	Positive (>128 U/ml)	Negative (<128 U/ml)	–

a *Calculated using the Chi-square test; NS, not significant*.

### Fil+ Individuals Exhibit Significantly Diminished Levels of IL-1α, IL-1β, IL-18, IL-12, and IL-23 and Significantly Enhanced Levels of IL-33 and IL-35

**Figure 1:**
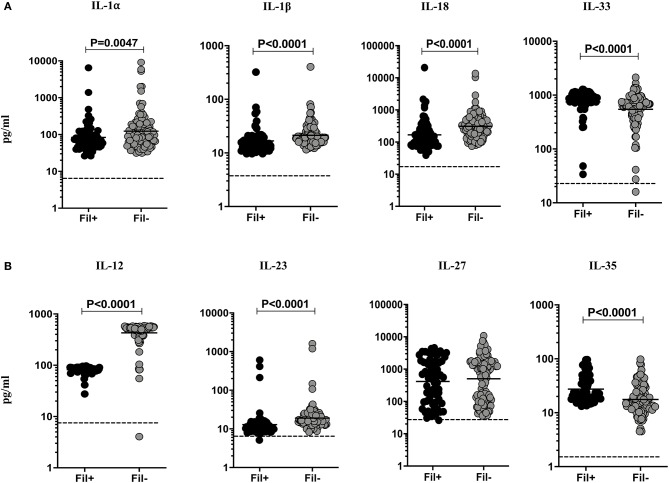
Altered plasma levels of IL-1 and IL-12 family of cytokines in filarial coinfected individuals with TBL. The plasma levels of **(A)** IL-1 (IL-1α, IL-1β, IL-18, and IL-33) and **(B)** IL-12 family (IL-12, IL-23, IL-27, and IL-35) of cytokines were measured in Fil+ (*n* = 67) and Fil– (*n* = 109) individuals with TBL. The results are represented as scatter plots with each circle representing a single individual. The bar indicates the geometric mean and the significant differences were calculated using non-parametric Mann-Whitney U test. The dotted line indicates the limit of detection for each cytokine.

Filarial infection is associated with diminished levels of pro-inflammatory cytokines in pulmonary TB (3). To assess the impact of coincident filarial infection on IL-1 family (IL-1α, IL-1β, IL-18, IL-33) and IL-12 family (IL-12, IL-23, IL-27, IL-35) of cytokines in TBL individuals, we measured the plasma levels of these cytokines (Figure 1). The plasma levels of IL-1α (Geometric mean (GM) of 83.82 vs. 123.4 pg/ml, *p* = 0.0047), IL-β (GM of 16.78 vs. 21.27 pg/ml, *p* < 0.0001) and IL-18 (GM of 167.7 vs. 310.0 pg/ml, *p* < 0.0001) were significantly lower in Fil+ compared to Fil– individuals. In contrast, the plasma levels of IL-33 (GM of 749.5 vs. 545.9 pg/ml, *p* < 0.0001) was significantly higher in Fil+ compared to Fil– individuals (Figure 1A). The plasma levels of IL-12 (GM of 82.12 vs. 428.3 pg/ml, *p* < 0.0001) and IL-23 (GM of 12.89 vs. 19.09 pg/ml, *p* < 0.0001) were significantly lower and the plasma levels of IL-35 (GM of 27.44 vs. 17.63 pg/ml, *p* < 0.0001) was significantly higher in Fil+ compared to Fil- individuals (Figure 1B). Hence, filarial coinfection is characterized by altered plasma levels of IL-1 and IL-12 family of cytokines.

### Fil+ Individuals Exhibit Significantly Diminished Levels of IL-2 and IL-9 and Significantly Enhanced Levels of IL-4

**Figure 2:**
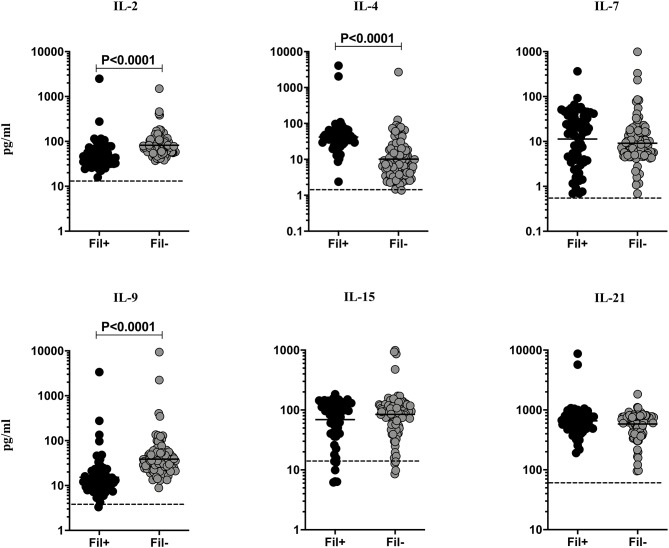
Altered plasma levels of γC cytokines in filarial coinfected individuals with TBL. The plasma levels of γC family (IL-2, IL-4, IL-7, IL-9, IL-15, and IL-21) of cytokines were measured in Fil+ (*n* = 67) and Fil– (*n* = 109) individuals with TBL. The results are represented as scatter plots with each circle representing a single individual. The bar indicates the geometric mean and the significant differences were calculated using non-parametric Mann-Whitney U test. The dotted line indicates the limit of detection for each cytokine.

Filarial infections are associated with alterations in the γc family of cytokines in pulmonary TB (3). Hence, we measured the circulating levels of γc family (IL-2, IL-4, IL-7, IL-9, IL-15, and IL-21) of cytokines to examine the influence of filarial coinfection among TBL individuals (Figure 2). The plasma levels of IL-2 (GM of 49.02 vs. 82.09 pg/ml, *p* < 0.0001) and IL-9 (GM of 15.26 vs. 38.79 pg/ml, *p* < 0.0001) were significantly lower and the plasma levels of IL-4 (GM of 41.93 vs. 10.18 pg/ml, *p* < 0.0001) was significantly higher in Fil+ compared to Fil– individuals. In contrast, there were no significant differences in the levels of IL-7, IL-15, and IL-21 between the two groups (Figure 2). Hence, filarial coinfection is characterized by altered plasma levels of γc family of cytokines.

### Fil+ Individuals Exhibit Significantly Enhanced Levels of IL-10 and IL-1Ra

**Figure 3:**
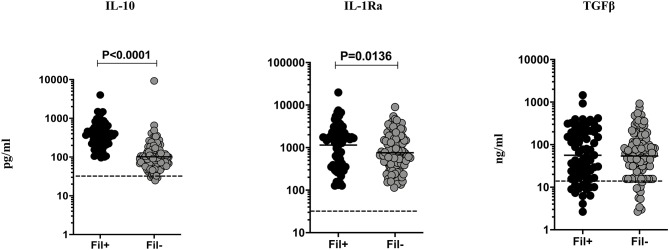
Elevated plasma levels of regulatory cytokines in filarial coinfected individuals with TBL. The plasma levels of regulatory (IL-10, IL-1Ra, and TGF-β) cytokines were measured in Fil+ (*n* = 67) and Fil– (*n* = 109) individuals with TBL. The results are represented as scatter plots with each circle representing a single individual. The bar indicates the geometric mean and the significant differences were calculated using non-parametric Mann-Whitney U test. The dotted line indicates the limit of detection for each cytokine.

Filarial infection is associated with elevated regulatory cytokines (IL-10 and TGF β) in pulmonary TB (3). Hence, we studied the influence of filarial coinfection on regulatory cytokines in TBL, by measuring the plasma levels of these cytokines (Figure 3). The plasma levels of IL-10 (GM of 366.9 vs. 102.0 pg/ml, *p* < 0.0001) and IL-1Ra (GM of 1148.0 vs. 755.7 pg/ml, *p* = 0.0136) were significantly higher in Fil+ compared to Fil– individuals. In contrast, there were no significant differences in the levels of TGF β between the two groups (Figure 3). Hence, filarial coinfection is characterized by increased plasma levels of regulatory cytokines.

### Fil+ Individuals Exhibit Significantly Enhanced Levels of CC Chemokines

**Figure 4:**
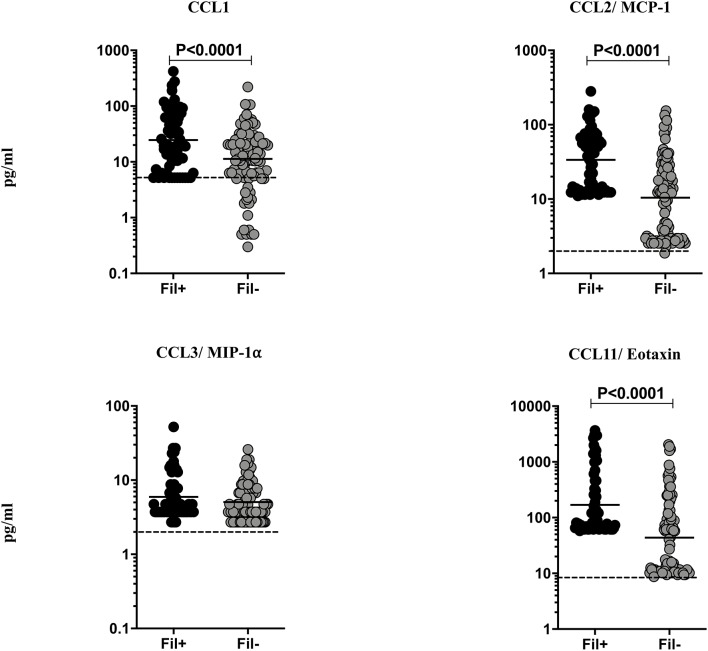
Elevated plasma levels of CC chemokines in filarial coinfected individuals with TBL. The plasma levels of CC (CCL1, CCL-2/MCP-1, CCL3/MIP1α, and CCL11/eotaxin) chemokines were measured in Fil+ (*n* = 67) and Fil– (*n* = 109) individuals with TBL. The results are represented as scatter plots with each circle representing a single individual. The bar indicates the geometric mean and the significant differences were calculated using non-parametric Mann-Whitney U test. The dotted line indicates the limit of detection for each chemokine.

Helminth infections are associated with diminished CC chemokines in latent TB (10). To study the association of filarial coinfection on CC chemokines in TBL, we measured the plasma levels of CCL1, CCL2, CCL3, and CCL11 (Figure 4). The plasma levels of CCL1 (GM of 24.64 vs. 11.31 pg/ml, *p* = 0.0024), CCL2 (GM of 33.59 vs. 10.43 pg/ml, *p* < 0.0001) and CCL11 (GM of 169.7 vs. 43.82 pg/ml, *p* < 0.0001) were significantly higher in Fil+ compared to Fil– individuals (Figure 4). Hence, filarial coinfection is characterized by increased plasma levels of CC chemokines.

### Fil+ Individuals Exhibit Significantly Enhanced Levels of Most CXC Chemokines

**Figure 5:**
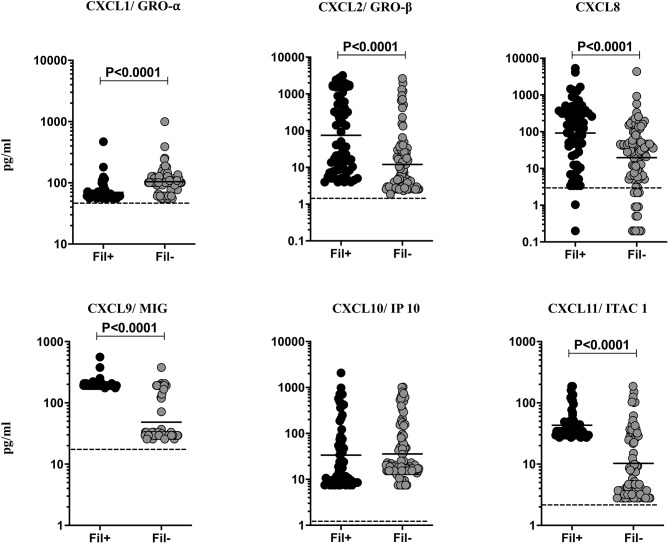
Elevated plasma levels of CXC chemokines in filarial coinfected individuals with TBL. The plasma levels of CXC (CXCL1/GRO-α, CXCL2/GRO-β, CXCL9/MIG, CXCL8, CXCL10/IP-10, CXCL11/ITAC1) chemokines were measured in Fil+ (*n* = 67) and Fil– (*n* = 109) individuals with TBL. The results are represented as scatter plots with each circle representing a single individual. The bar indicates the geometric mean and the significant differences were calculated using non-parametric Mann-Whitney U test. The dotted line indicates the limit of detection for each chemokine.

Helminth infections are associated with diminished CXC chemokines in latent TB (10). To study the association of filarial coinfection on CXC chemokines in TBL, we measured the systemic levels of CXCL1, CXCL2, CXCL8, CXCL9, CXCL10, and CXCL11 (Figure 5). The plasma levels of CXCL2 (GM of 74.75 vs. 12.04 pg/ml, *p* < 0.0001), CXCL8 (GM of 92.34 vs. 19.64 pg/ml, *p* < 0.0001), CXCL9 (GM of 199.4 vs. 48.40 pg/ml, *p* < 0.0001) and CXCL11 (GM of 43.07 vs. 10.27 pg/ml, *p* < 0.0001) were significantly higher and plasma levels of CXCL1 (GM of 69.71 vs. 104.1 pg/ml, *p* < 0.0001) was significantly lower in Fil+ compared to Fil– individuals (Figure 5). Hence, filarial coinfection is characterized by increased plasma levels of most CXC chemokines.

## Discussion

Extra-pulmonary TB accounts for ~20% of all TB cases and lymph nodes are often the most common site (4). Previous studies have shown that helminth infections can influence the pathogenesis and immune response to pulmonary TB (3, 11). To our knowledge, our study is the first to examine the bacteriological and immunological outcomes in filaria-TBL coinfections and the second to examine the same in helminth-TBL coinfections in total (12). Our previous study had demonstrated that *Stronglyoides stercoralis* coinfection could modulate cytokine (but not chemokine) responses both systemically and in an antigen—specific manner (12). We had also demonstrated elevated bacterial burdens in that helminth-TBL coinfection (12). We expand our data on those findings and report the influence of filarial coinfection on the cytokine and chemokine response in TBL disease. We also validate our previous report that co-infected helminth infection is associated with higher bacterial burdens in the affected lymph node of TBL individuals. This study offers new insights into the pathogenesis of TBL disease in the presence of co-prevalent helminth infections and suggests that treatment of helminth infections could influence the outcome of TBL disease.

Among cytokines involved in host immunity to TB disease, the IL-1 and IL-12 family of cytokines play a major role. IL-1α, IL-1β, and IL-18 are known to be essential for immunity to TB infection (13, 14). Both IL-1 cytokines signal through the same IL-1 receptor and are known to be independently required for host resistance and deficiency in either cytokine renders mice more susceptible to TB infection (15). Similarly, IL-18 is important for host immunity and functions by induction of IFNγ (16). Finally, TBL is characterized by diminished plasma and TB—antigen stimulated levels of IL-1β and IL-18 (17). Our data therefore suggest that in the presence of filarial infection, protective cytokines are dampened in the circulation of TBL individuals. In contrast, IL-33 is a predominantly anti-inflammatory cytokine belonging to the IL-1 family (18). It is mostly expressed in non-hematopoietic cells like adipocytes, fibroblasts, intestinal and bronchial epithelial cells and endothelial cells (19–21). IL-33 is also expressed by hematopoietic cells mainly by dendritic cells (DCs) to lesser extent (22). IL-33 acts an alarmin in the induction of Type 2 cytokine responses (23) and is known to help attenuate ongoing TB infection in mice (24). Since IL-33 is induced in a variety of Th2 settings, it is not surprising to see that IL-33 levels were enhanced in filarial coinfected individuals. However, whether IL-33 has a beneficial or detrimental effect on the TBL disease remains to be determined.

IL-12 and IL-23 are the two primary cytokines fundamentally important in resistance to TB infection as demonstrated both by animal models and human immune deficiencies (25–30). IL-12 by its induction of Type 1 responses and IL-23 by driving Type 17 cytokine responses are critical in host immunity to TB. Our data showing diminished levels of both the cytokines also suggest that filarial infections modulate immunity in TBL by decreasing Type 1 and Type 17 cytokine responses. IL-27 has an important role in immunity to TB (31) but is not modulated in the presence of coinfection. Finally, IL-35 is an anti-inflammatory cytokine of the IL-12 family (32), whose role in TB is not clear. Our data suggest that filarial modulation of TBL disease results in higher levels of IL-35, which might be potentially host-detrimental. The role of γc cytokines in host immunity to TB infection and disease is not well understood. IL-2 and IL-21 have been shown to be important to host resistance in animal models (33, 34) and regulation of these cytokines has been demonstrated in human pulmonary TB (35). Our data on the modulation of γc cytokines in the presence of filarial coinfection suggests that IL-2 and IL-9 (but not IL-7, IL-15 or IL-21) levels are significantly diminished. The implication of the alterations in IL-2 and IL-9 needs to be examined further.

One potential mechanism for the decrease in baseline levels of proinflammatory cytokines in TBL individuals with filarial coinfection could be a concomitant increase in regulatory cytokines. Our data suggest that IL-4, IL-10, and IL-1Ra are significantly increased in Fil+ individuals suggesting a plausible biological mechanism for the downmodulation of IL-1 and IL-12 family of cytokines. Also, since IL-4, IL-10, and IL-1Ra are known to play a detrimental role in enhancing susceptibility to infection (36–38), our data indicate that alteration of the balance between protective and regulatory cytokines plays a pivotal role in the establishment of cytokine responses in filarial-TBL coinfection. In addition, this balance could also be impacted by the elevated levels of the anti-inflammatory cytokines—IL-33 and IL-35. Interestingly, no difference in TGFβ levels was observed between coinfected and uninfected individuals in TBL disease.

Chemokines play an important role in cell migration to TB infected organs and are critical for TB control (8, 9). Indeed, productive granuloma formation is known to be tightly regulated by chemokines (8, 9). However, chemokine dysregulation can shift the balance from protection to inflammation and pathology in TB disease (8, 9). We have previously shown that plasma chemokines are markers of disease severity, bacterial burdens and delayed culture conversion in pulmonary TB (39). Our data clearly show that both CC (CCL1, CCL2, CCL11) and CXC (CXCL2, CXCL8, CXCL9, CXCL11) are clearly elevated in filarial-TBL coinfection. Due to their ability to recruit neutrophils, eosinophils and other inflammatory cell types (40), these chemokines could play a potentially pathogenic role in TBL. Finally, dysregulation of chemokine production has been shown to enhance TB cavitation and pathology and therefore, excessive circulating levels of chemokines could also contribute to this process (41).

Our study suffers from the limitation of being cross-sectional, lacking the ability to attribute cause-effect mechanisms, measuring only plasma (and not local) levels of cytokines and chemokines and not performing stool examinations for other helminth infections. Nevertheless, our study clearly shows that coexistent filarial infection has major effects on TBL bacteriology and immunology. In addition to being associated with higher bacterial burdens, filarial infections appear to downmodulate the systemic levels of key protective cytokine families and upregulate the systemic levels of key pathogenic cytokines and chemokines. Thus, by its ability to influence the cytokine and chemokine milieu in TBL, coexistent filarial infection imparts certain deleterious effects in the course of TBL disease. The role of immune cell subsets in helminth—TBL coinfection is currently under investigation. Overall, our results suggest the crucial role of filarial coinfection in modulating the necessary protective cytokines and chemokines in TBL disease. Therefore, our future approach is to study the association of those cytokines and chemokines in those groups of study individuals after the completion of anthelmintic treatment to understand the effect of anthelmintic treatment on this important component of the immune response against the TB pathogen.

## Materials and Methods

### Ethics

All individuals (participants above 18 years of age were enrolled in the study) were assessed as part of a natural history study protocol approved by Institutional Review Boards of National Institute for Research in Tuberculosis (NIRT, NIRTIEC2010002) and informed written consent was obtained from all participants involved in the study.

### Study Population

We studied a group of 176 individuals with TBL, 67 of whom were infected with filarial infection (hereafter Fil+) and 109 of whom were negative for filarial infection and only had TBL (hereafter Fil–) (Table 1). This was a nested case control study within a larger study on the immune responses in pulmonary and extrapulmonary TB conducted at the Government Stanley Medical Hospital, Chennai. Out of 252 extrapulmonary TB cases, 176 TBL cases were used for this study. TBL diagnosis was made on the basis of excision biopsy (i.e., affected lymph nodes) showing culture positivity for *M. tuberculosis*. Culture was performed using homogenized lymph node tissue and culture grades [1+ (1–20 colonies)/2+ (20–100 colonies)/3+(>100 colonies)] were used to identify the bacterial burdens as ascertained by growth of *M. tuberculosis* on Lowenstein-Jensen solid media [11]. Culture scoring was done independently and blinded to the filarial infection status. Filarial (*Wuchereria bancrofti*) infection was diagnosed by assessing the filarial (both microfilaremic and amicrofilaremic) antigen levels using TropBio Og4C3 enzyme-linked immunosorbent assay (ELISA) (Trop Bio Pty. Ltd., Townsville, Queensland, Australia). The cut off range to detect filarial antigen is >128 antigen unit (U/ml) and considered as positive and <128 (U/ml) is considered as negative for the presence of filarial antigen. All the study individuals were BCG vaccinated, negative for HIV and not under any steroid treatment. We did not perform any stool microscopy examination for these individuals and they were anti-tuberculosis and anthelmintic treatment naive. All these individuals were from a peri-urban area in Chennai, India.

### Plasma Collection and Measurement of Cytokines by ELISA

Blood samples (10 ml) were collected in sodium heparin tubes and plasma was collected by centrifugation at 2,600 revolutions per minute (rpm) for 10 min at 4°C and stored at −80°C. The following cytokines-IL-1α, IL-Ra, IL-1β, IL-2, IL-4, IL-7, IL-9, IL-10, IL-12, IL-15, IL-18, IL-21 (DuoSet R&D Systems), IL-23, IL-27, IL-33, IL-35 (e-Biosciences) and TGFβ (BioLegend) were measured using ELISA.

### Measurement of Chemokines by Multiplex Immunoassay

Plasma chemokine levels were measured using a multiplex immunoassay system (Luminex, Biorad) with a kit from R&D Systems. The chemokines measured were: CC (CCL1, CCL2/MCP-1, CCL3/MIP1α, CCL4/MIP-1β, CCL11/eotaxin) and CXC (CXCL1/GRO-α, CXCL2/GRO-β, CXCL9/MIG, CXCL8/IL-8, CXCL10/IP-10, CXCL11/ITAC1).

### Statistical Analysis

All the data were analyzed using GraphPad PRISM (GraphPad Software, Inc., San Diego, CA, USA) tool. Geometric means (GM) were used for measurements of central tendency and nonparametric Chi-square test was used to compare statistically significant differences in age, gender and bacterial burdens. Mann-Whitney U test was used to compare the statistically significant differences among the cytokines and chemokines analyzed.

## Data Availability Statement

All datasets generated for this study are included in the article/supplementary material.

## Ethics Statement

The studies involving human participants were reviewed and approved by Institutional Review Boards of National Institute for Research in Tuberculosis (NIRT, NIRTIEC2010002). The patients/participants provided their written informed consent to participate in this study.

## Author Contributions

GK and SB: conceived and designed the experiments, analyzed the data, and wrote the paper. GK and SM: performed the experiments. VB, DN, RS, and SB: contributed materials, reagents, analysis tools.

## Conflict of Interest

The authors declare that the research was conducted in the absence of any commercial or financial relationships that could be construed as a potential conflict of interest.
